# Evaluation of Antihyperglycemic Activity of *Citrus limetta* Fruit Peel in Streptozotocin-Induced Diabetic Rats

**DOI:** 10.5402/2011/869273

**Published:** 2011-07-21

**Authors:** Sriparna KunduSen, Pallab K. Haldar, Malaya Gupta, Upal K. Mazumder, Prerona Saha, Asis Bala, Sanjib Bhattacharya, Biswakanth Kar

**Affiliations:** ^1^Department of Pharmaceutical Technology, Jadavpur University, Kolkata, West Bengal 700032, India; ^2^Guru Nanak Institute of Pharmaceutical Science and Technology, Panihati, Kolkata, West Bengal 700114, India; ^3^Pharmacognosy Division, Bengal School of Technology, Sugandha, Hooghly, West Bengal 712102, India

## Abstract

The present paper aims to evaluate antihyperglycemic activity of methanol extract of *Citrus limetta* fruit peel (MECL) in streptozotocin-induced (STZ; 65 mg/kg b.w.) diabetic rats. Three days after STZ induction, diabetic rats received MECL orally at 200 and 400 mg kg^−1^ body weight daily for 15 days. Glibenclamide (0.5 mg kg^−1^ p. o.) was used as reference drug. Blood glucose levels were measured on 0th, 4th, 8th, and 15th days of study. Serum biochemical parameters namely, SGOT, SGPT and ALP were estimated. The TBARS and GSH levels of pancreas, kidney, and liver were determined. MECL significantly (*P* < 0.001) and dose dependently normalized blood glucose levels and serum biochemical parameters, decreased lipid peroxidation, and recovered GSH as compared to those of STZ control. The present paper infers that in STZ-induced diabetic Wistar rats, *C. limetta* fruit peel demonstrated a potential antihyperglycemic effect which may be attributed to its antioxidant property.

## 1. Introduction

Diabetes mellitus (DM) is a chronic disease which is caused either by inherited disability or acquired deficiency in production of the hormone insulin and its subsequent inability to regulate the blood glucose level and also where there is sufficient production of insulin but the insulin secreted is unable to regulate the blood glucose levels. The outcome of the above two conditions is that there is an increased level of blood glucose which in turn damages many of the vital organs (kidney, eye, etc.) of the body [1]. There is increasing demand by patients to use natural products with antidiabetic activity due to side effects associated with the use of insulin and oral hypoglycemic agents [2–4]. Various traditional medicinal plants reported to have proven hypoglycaemic properties such as *Allium sativum *(Garlic), *Azadirachta indica *(Neem), *Vinca rosea *(Vinca), *Trigonella foenum-graceum *(Fenugreek), *Momordica charantia *(Bitter gourd), and *Ocimum scantum *(Tulsi). Citrus fruits have been recommended in traditional herbal medicine as the source of diabetic medication or remedy for diabetes [5]. Citrus fruits are abundant in the Indian subcontinent. Lemon, lime, pomelo, sweet lime, and orange, and so forth are cultivated in abundance in different regions of India. The citrus fruits are well recognized for their various ethnomedicinal uses. These properties are attributed to their flavonoids and limonoids which are proven to posses anti-inflammatory and antitumor effects [6, 7]. The peels are rich in pectin which is known to possess blood sugar lowering and cholesterol lowering properties [8]. Other than these, carotenoids and hydroxycinnamic acids are also abundant in citrus peels [9, 10]. Hydroxycinnamic acid compounds inhibit the oxidation of low-density lipoproteins [11] and have anticancer [12] and antimicrobial activities [13]. A high carotenoid intake also decreases the risk of cancer, cataracts, and cardiovascular diseases [14].

According to Andrade-Citto (1995),* Citrus limetta* Risso (Rutaceae) is known to be an antihyperglycemic plant in Mexican folklore [5], *C. limetta* fruits and leaves are used for common cold [15]; decreasing cholesterol level, fever regulation, regulating inflammation, digestive disorders, and so forth as well as blood pressure modulator [16]. Flavonoids hespiridin and naringin are found to be present in the peel and inner part of the fruit of *Citrus limetta* [17]. The essential oils *α*-pinene, *β*-pinene, sabinene, *β*-myrcene, *p*-cymene, limonene, *γ*-terpinene, neryl acetate, *β*-bisabolene, and *α*-bergamotene were isolated from the zests of *Citrus limetta* Risso [18]. Limonene, *γ*-terpinene was isolated from the peel, and their minimum inhibitory concentration (MIC) and maximal tolerated concentration (MTC) were estimated against *Pseudomonas putida* [19].

The leaf extract of this plant was evaluated for its antagonistic activity on the hypertensive action of angiotensin II [20]. The fruit has shown anti-inflammatory and antithrombotic actions [21, 22]. Present study aimed to explore the antihyperglycemic potential of *Citrus maxima *leaf against streptozotocin-induced diabetic rats.

## 2. Materials and Methods

### 2.1. Plant Material

*C. limetta* fruits were collected from Nadia region of West Bengal, India in the months of March-April, 2007. The plant material was authenticated by the Botanical Survey of India, Shibpur, Howrah, and the voucher specimen (PMU-2/JU/2007). It is stored in our laboratory for further references.

### 2.2. Extraction

Fresh fruit peels of *C. limetta* were taken and grounded, and about 500 g of the plant material was consecutively macerated for seven days each in petroleum ether, ethyl acetate, chloroform, and methanol, respectively. On basis of the preliminary phytochemical tests conducted, the methanol extract was found to be rich in terms of chemical constituents and therefore was selected for the hypoglycaemic activity. The methanol was removed under reduced pressure to obtain a semisolid mass (MECL, yield 10.56% w/w). The MECL was then stored in vacuum desiccator until used. The plant extract was subjected to screening for various phytochemicals. The presence of steroids, alkaloids, tannins, flavonoids, glycosides, and so forth was established in the MECL [23].

### 2.3. Chemicals

Glibenclamide tablet (DaonilTM, Hoechst, India), streptozotocin (Himedia, Mumbai, India), 5,5-dithio bis-2 nitro benzoic acid (DTNB), reduced glutathione (Sisco Research Lab, Mumbai, India), thiobarbituric acid (TBA), trichloroacetic acid (TCA), and nitroblue tetrazolium (NBT) were used. All the other reagents used were of AR grade.

### 2.4. Animals

Six- to eight-week-old male Wistar albino rats (180 ± 20 g) were obtained from Ghosh and Co., Kolkata. They were acclimatized to the laboratory conditions prior to the study for seven days. The animals were kept at 25 ± 2°C and a relative humidity of 40–45% with alternative day and night cycles of 12 hours each. The animals had free access to pellet food (Hindustan Lever, Mumbai, India) and water *ad libitum*.

### 2.5. Acute Toxicity

The acute toxicity of the extract was determined according to the OECD guideline no. 420 [24]. Male albino mice weighing 27–30 g were used for this study. MECL was given to four groups (*n* = 5) of animals at 5, 50, 300, and 2000 mg/kg b.w. p. o. The treated animals were under observation for 14 days, for mortality and general behaviour. No death was observed till the end of the study. The test samples were found to be safe up to the dose of 2000 mg/kg b.w.

### 2.6. Effect on Glucose Tolerance in Rats

The animals were divided into four groups. Group I served as negative control group (0.9% NaCl, p. o). Group II and III were treated with MECL at dose levels 200 and 400 mg kg^−1^ b.w. p. o., respectively. Group IV was treated with 0.5 mg kg^−1^ glibenclamide p. o. Zero hour-blood sugar levels were determined on 18-hour fasted animals. After 30 min of drug treatment the animals were fed with glucose (5 g^−1^ kg) and blood glucose was determined after 30 minutes, 1, 2, and 3 h of the glucose load [25]. The blood sugar level was measured using “Accu-chek Active” Test strip in Accu-chek Active Test meter.

### 2.7. Oral Hypoglycaemic Activity in Normal Rats

Healthy male Wistar albino rats weighing 180 ± 20 g were selected for the study. The animals used for the study were fasted for twelve hours. The animals were classified into four groups (*n* = 6). Group I served as saline control (5 mL kg^−1^ b.w. 0.9% NaCl p. o.); Groups II and III were treated with MECL at dose levels 200 and 400 mg kg^−1^ b.w. p. o. Group IV was treated with glibenclamide (0.5 mg kg^−1^ p. o.). Blood samples were collected from the tail tip at 0 (before oral administration) and 2 hours 30 minutes after drug administration. The blood sugar level was measured using “Accu-chek Active” Test strip in Accu-chek Active Test meter.

### 2.8. Induction of Experimental Diabetes

Streptozotocin was administered to 18-hour fasted rats and observed for 5 days. STZ was freshly dissolved in citrate buffer (0.01 M, pH 4.5). The injection volume was prepared to contain 65 mg mL^−1^ and injected as 0.1 mL 100 g^−1^ of body weight [26]. After 5 days the fasting blood glucose level was measured on overnight fasted rats by using “Accu-chek Active” Test strip in Accu-chek Active Test meter. The animals that developed blood glucose level of more than 250 mg dL^−1^ were selected for the study.

### 2.9. Treatment of Diabetic Animals

Thirty male Wistar albino rats (160–200 g) were divided into five groups (*n* = 6) out of which six normal animals were kept as nondiabetic saline control. Group II served as diabetic control. Group III and IV received MECL (200 and 400 mg kg^−1^ b.w. p. o.) and Group V received reference drug glibenclamide (0.5 mg kg^−1^ b.w. p. o.) daily for 15 days, respectively [27].

### 2.10. Sample Collection and Biochemical Studies

Fasting blood sugar of the animals was determined on 0th, 5th, 8th, and 15th day collected from the tail vein. On 15th day blood was collected from the retro orbital plexus of the eye for the determination of SGPT, SGOT, and ALP. All the animals from different groups were sacrificed on the 15th day and their liver, kidney, and pancreas were isolated for the estimation of TBARS and GSH. Thiobarbituric acid reactive substances (TBARS) were estimated in the liver and kidney by the method of Fraga et al. (1981) and are expressed as mmol 100 g^−1^ of tissue [28]. Reduced glutathione (GSH) was determined by the method of Ellman [29], and the GSH activity is expressed as mg 100 g^−1^ of tissue [29].

### 2.11. Statistical Analysis

All the values of body weight, fasting blood sugar, and biochemical parameters were expressed as mean ± standard error of mean (SEM) and were analysed for ANOVA and Dunnett's “*t*” test. The values of *P* < 0.001 were considered statistically very significant and *P* < 0.05 were considered as significant.

## 3. Results

Acute toxicity study was carried out on Swiss albino mice which showed that MECL was safe up to 2000 mg kg^−1^ b.w. p. o. Preliminary phytochemical study indicated the presence of flavonoids, alkaloids, tannins, and saponin in MECL. 

The effect of MECL in lowering the blood sugar level in glucose-overloaded rats is summarized in Table 1. MECL-treated groups have shown significant (*P* < 0.05) decrease in the blood sugar level of the glucose overloaded rats.

The effect of MECL in lowering of blood sugar levels of normal rats are summarized in Table 2. The MECL-treated groups show significant reduction in the blood sugar levels as compared to the reference drug in a dose-dependant manner. 

The measured blood glucose levels of nondiabetic and STZ-induced diabetic rats are shown in Table 3. Administration of MECL in STZ-induced diabetic rats, at the doses of 200 and 400 mg kg^−1^ b.w., produced a significant reduction in blood glucose levels when compared with the STZ-control group. 

The body weights of normal and diabetic rats are summarized in Table 4. The final body weights were significantly decreased in the STZ control group when compared with the saline control group. The table exhibited an improvement of body weight after treatment with the extract when compared to that of the STZ control group. 

In case of biochemical estimation, improvement of serum enzyme levels were observed in the treated groups with respect to the diabetic control group as shown in Table 5. The level of TBARS and GSH activities in pancreatic, kidney, and liver tissues of experimental diabetic rats are shown in Table 6. There was a significant elevation of lipid peroxide in the pancreas, liver, and kidney with diabetes when compared to the saline control group. It was seen that the administration of MECL helped to decrease pancreatic, hepatic, and renal TBARS levels, which is an indication of the inhibition of oxidative damage of the said tissues. There was also a significant decrease in the level of GSH in the STZ control group when compared with the saline control group. Administration of MECL at the doses of 200 and 400 mg kg^−1^ b.w. increased the GSH level in the liver and kidney of STZ-induced rats.

## 4. Discussion

Streptozotocin (STZ) is widely used for the induction of diabetes mellitus in experimental animals. It is postulated to induce diabetes by the degeneration and necrosis of *β* cells of islet of langerhans of pancreas, which leads to the reduction in insulin release [30]. The ability of STZ to selectively enter the *β* cells *via *the low-affinity glucose transporter GLUT2 in the plasma membrane can be attributed to the presence of glucose moiety in it. After STZ enters the cells it gets converted to reactive methylcarbonium ions that alkylate DNA and induce free radical generation which target the DNA sugar moiety and result in DNA strand breakage [31].

In the present investigation it was observed that oral administration of the MECL at the doses of 200 and 400 mg kg^−1^ b.w. produced effective hypoglycemic and antihyperglycemic activity in normoglycemic, glucose overloaded, as well as STZ-induced diabetic rats. MECL also possessed a significant controlling effect in the loss of body weight that occurred in diabetic rats. The food intake of the STZ control decreased significantly. For all other groups food intake was comparable to that of saline control.

Preliminary phytochemical analysis of MECL indicated the presence of flavonoids. Flavonoids are polyphenolic compounds that are well-known antioxidants because of their electron-donating properties, either scavenging the principal propagating radicals or halting the radical chain. *In vitro *free radical scavenging activity of MECL was performed by the authors, and the extract was found to have significant free radical scavenging activity when tested against different free radicals [32].

Elevated levels of the serum enzymes (SGPT, SGOT, and ALP) in the diabetic control group reflect the significant alteration of liver function by STZ induction [33]. Treatment with MECL restored the elevated enzyme levels to normal level in a dose-dependent manner (*P* < 0.001). 

Under normal physiological conditions, the human body can compensate for a mild degree of oxidative stress, and remove oxidatively damaged molecules by activating antioxidant enzymes. These antioxidants are able to resist oxidative stress by scavenging free radicals, inhibiting lipid peroxidation, and increasing glutathione [33].

Lipid peroxidation is a natural phenomenon involved in peroxidative loss at unsaturated lipids, thus bringing about lipid degradation and membrane disorganization. Peroxidized lipid has been considered to play a significant role in the pathogenesis of several diseases, and may be taken as a molecular mechanism of cell injury under pathological conditions. Lipid peroxidation is usually measured through its catabolite malondialdehyde (MDA) in terms of TBARS (thiobarbituric acid reactive substances) as a marker of oxidative stress [33]. In the current study it was observed that there was a significant decrease in the TBARS levels of the MECL-treated rats in comparison to that of the diabetic control group.

Glutathione (GSH) is one of the abundant tripeptide nonenzymatic biological antioxidants present in the liver and kidney. GSH plays multifunctional role in antioxidant defence. It is a direct scavenger of free radicals as well as a cosubstrate for peroxide detoxification by glutathione peroxidases [34]. It is postulated that a decrease in tissue GSH is due to either decreased synthesis or increased degradation of GSH by oxidative stress [35]. In the current study it is observed that MECL was able to decrease elevated levels of GSH in comparison to the diabetic control rats.

The present study shows that the methanol extract of the fruit peels of *C. limetta* has potent antihyperglycemic activity against STZ-induced diabetes as well as having hypoglycemic activity in normoglycemic rats and in glucose overloaded rats. The *Citrus* plants are rich in flavonoids which are polyphenolic compounds having potent antioxidant property. 

As discussed earlier in the introduction section, the fruit peel of *Citrus limetta* contains the flavonoids hesperidin and naringin. Hesperidin and naringin both are proven to be potent hypoglycaemic agents, and their hypoglycaemic activity is postulated to be partly mediated by hepatic glucose-regulating enzymes in C57BL/KsJ-db/db mice [36]. Dietary hesperidin also exerts hypoglycemic and hypolipidemic effects in streptozotocin-induced diabetic rats [37]. Naringin provided a significant amelioration of hypoglycaemic and antioxidant activity in STZ-induced diabetic rats [38]. Therefore, it can be postulated that the presence of flavonoids in the extract might be the reason of the antihyperglycemic action shown by MECL. Further investigation in this line can pinpoint the exact mechanism of action of *Citrus limetta.* In the future identifying the active biological principle of *C. limetta* may provide novel, safe antidiabetic compound.

## Figures and Tables

**Table 1:** Effect of MECL in glucose-loaded hyperglycemic rats.

Treatments	Dose	Blood glucose concentration (mg/dL)		
		0 h	1 h	2 h
Control	—	82.3 ± 1.4	183.7 ± 7.4	134.7 ± 10.7
MECL	200 mg/kg (p.o.)	77.3 ± 5.2*	151.7 ± 3.8*	93.3 ± 2.8*
MECL	400 mg/kg (p.o.)	83.4 ± 2.4*	102.4 ± 5.6*	84.7 ± 2.7*

Values are expressed as mean ± SEM (*n* = 6); *P < 0.05 when compared with control group.

**Table 2:** Hypoglycemic activity of different doses of MECL in normal rats.

Group	Dose	Blood glucose level mg/dL	
		0th hour	2 hour 30 min
Saline control	5 mL/kg (p.o.)	79.62 ± 4.4	78.66 ± 1.4
MECL	200 mg/kg (p.o.)	96.1 ± 1.01*	87.7 ± 2.2*
MECL	400 mg/kg (p.o.)	97.3 ± 1.2*	65.7 ± 4.2*
Glibenclamide	0.5 mg/kg (p.o.)	82.5 ± 1.4*	77.83 ± 2.5*

Values are expressed as mean ± SEM (*n* = 6); *P < 0.001 compared with saline control group.

**Table 3:** Effect of different doses of MECL on blood sugar level of normal and hyperglycaemic rats.

Group	Dose	Blood glucose level mg/dL			
		0th day	4th day	8th day	15th day
Saline control	5 mL/kg (p.o.)	79.62 ± 4.4	78.23 ± 4.2	78.16 ± 2.4	78.12 ± 3.1
STZ control	65 mg/kg (i.p.)	267.8 ± 12.3*	288.7 ± 15.6*	287.3 ± 6.3*	294.8 ± 2.8*
STZ + MECL	200 mg/kg (p.o.)	266.5 ± 4.6**	100.6 ± 4.4**	74.9 ± 5.8**	74.2 ± 2.7**
STZ + MECL	400 mg/kg (p.o.)	246.2 ± 3.2**	93.8 ± 2.9**	81.42 ± 2.7**	79.3 ± 3.02**
STZ + Glibenclamide	0.5 mg/kg (p.o.)	281.2 ± 0.3**	96.3 ± 4.6**	77.83 ± 6.9**	74.7 ± 1.7**

Values are expressed as mean ± SEM (*n* = 6); *P < 0.001 compared with saline control group and **P < 0.001 compared with STZ-control group.

**Table 4:** Effect on body weight of different doses of MECL in normal and hyperglycaemic rats.

Group	Dose	Body weight in grams	
		0th day	15 th Day
Saline control	5 mL/kg (p.o.)	176.16 ± 7.51	197 ± 5.8
STZ control	65 mg/kg (i.p.)	174.17 ± 1.3*	116.25 ± 1.9*
STZ + MECL	200 mg/kg (p.o.)	185.8 ± 1.7**	142 ± 1.4**
STZ + MECL	400 mg/kg (p.o.)	171.7 ± 2.1**	140.8 ± 1.1**
STZ + Glibenclamide	0.5 mg/kg (p.o.)	182.32 ± 6.4**	173.23 ± 9.5**

Values are expressed as mean ± SEM (*n* = 6); *P < 0.001 compared with saline control group and **P < 0.001 compared with STZ-control group.

**Table 5:** Effect on different serum enzyme levels of different doses of MECL in normal and hyperglycaemic rats.

Group	Dose	SGOT (IU/L)	SGPT (IU/L)	ALP (KA units)
Saline control	5 mL/kg (p.o.)	130.5 ± 1.3	97.96 ± 0.15	28.33 ± 1.76
STZ control	65 mg/kg (i.p.)	242.6 ± 1.21*	206.7 ± 0.14*	73.01 ± 1.81*
STZ + MECL	200 mg/kg (p.o.)	177.04 ± 0.8**	131.3 ± 0.14**	47.3 ± 1.2**
STZ + MECL	400 mg/kg (p.o.)	158.79 ± 0.84**	125.74 ± 0.73**	43.2 ± 2.4**
STZ + Glibenclamide	0.5 mg/kg (p.o.)	143.38 ± 2.26**	119.5 ± 1.22**	37.21 ± 1.65**

Values are expressed as mean ± SEM (*n* = 6); *P < 0.001 compared with saline control group and **P < 0.001 compared with STZ-control group.

**Table 6:** Effect of MECL on different tissue enzymes of normal and hyperglycaemic rats.

Group	Dose	Lipid peroxidation (mM 100 g^−1^ of tissue )			Glutathione (mg 100 g^−1^ of tissue)		
		Pancreas	Liver	Kidney	Pancreas	Liver	Kidney
Saline control	5 mL/kg (p.o.)	1.14 ± 0.08	1.02 ± 0.05	1.49 ± 0.1	34.56 ± 3.2	49.3 ± 2.1	23.6 ± 1.3
STZ control	65 mg/kg (i.p.)	1.8 ± 0.09*	1.85 ± 0.09*	2.34 ± 0.6*	13.7 ± 1.4*	25.9 ± 1.5*	6.4 ± 2.4*
STZ + MECL	200 mg/kg (p.o.)	1.4 ± 0.02**	1.4 ± 0.08**	1.7 ± 0.08**	41.09 ± 0.024**	27.4 ± 0.62**	18.86 ± 0.16**
STZ + MECL	400 mg/kg (p.o.)	1.31 ± 0.09**	1.12 ± 0.07**	1.62 ± 0.02**	36.08 ± 0.05**	31.75 ± 0.031**	17.14 ± 0.29**
STZ + Glibenclamide	0.5 mg/kg (p.o.)	1.23 ± 0.07**	1.07 ± 0.02**	1.51 ± 0.06**	41.31 ± 2.3**	41.78 ± 2.1**	19.61 ± 1.5**

Values are expressed as mean ± SEM (*n* = 6); *P < 0.001 compared with saline control group and **P < 0.001 compared with STZ-control group.
